# Dosage of the *Abcg1-U2af1* Region Modifies Locomotor and Cognitive Deficits Observed in the Tc1 Mouse Model of Down Syndrome

**DOI:** 10.1371/journal.pone.0115302

**Published:** 2015-02-23

**Authors:** Damien Marechal, Patricia Lopes Pereira, Arnaud Duchon, Yann Herault

**Affiliations:** 1 Institut de Génétique et de Biologie Moléculaire et Cellulaire, Illkirch, 1 rue Laurent Fries, 67404 Illkirch, France; 2 Centre National de la Recherche Scientifique, UMR7104, Illkirch, France; 3 Institut National de la Santé et de la Recherche Médicale, U964, Illkirch, France; 4 Université de Strasbourg, Illkirch, France; 5 Transgenese et Archivage Animaux Modèles, TAAM, CNRS, UPS44, 3B rue de la Férollerie 45071 Orléans, France; 6 Institut Clinique de la Souris, ICS, 1 rue Laurent Fries, 67404 Illkirch, France; CNRS UMR7275, FRANCE

## Abstract

Down syndrome (DS) results from one extra copy of human chromosome 21 and leads to several alterations including intellectual disabilities and locomotor defects. The transchromosomic Tc1 mouse model carrying an extra freely-segregating copy of human chromosome 21 was developed to better characterize the relation between genotype and phenotype in DS. The Tc1 mouse exhibits several locomotor and cognitive deficits related to DS. In this report we analyzed the contribution of the genetic dosage of 13 conserved mouse genes located between *Abcg1* and *U2af1*, in the telomeric part of Hsa21. We used the Ms2Yah model carrying a deletion of the corresponding interval in the mouse genome to rescue gene dosage in the Tc1/Ms2Yah compound mice to determine how the different behavioral phenotypes are affected. We detected subtle changes with the Tc1/Ms2Yah mice performing better than the Tc1 individuals in the reversal paradigm of the Morris water maze. We also found that Tc1/Ms2Yah compound mutants performed better in the rotarod than the Tc1 mice. This data support the impact of genes from the *Abcg1-U2af1* region as modifiers of Tc1-dependent memory and locomotor phenotypes. Our results emphasize the complex interactions between triplicated genes inducing DS features.

## Introduction

Down syndrome (DS; OMIN #190685) is a multigenic disorder, resulting from three copies of human chromosome 21 (Hsa21) [1]. This condition is a paradigm of human aneuploid disorders with a direct consequence of gene dosage [2–4] and a general perturbation of whole transcriptome [5]. DS represents one-third of cases of intellectual disabilities and cognitive impairment in school-aged children [6–8] and is associated with a wide range of dysmorphologies, such as characteristic faces, skeletal anomalies and brain alterations at the prefrontal cortex, hippocampus and cerebellum levels [9,10]. Clinical features of DS also include developmental delay, metabolic defects, other symptoms and associated diseases but their overall expressivity and penetrance are highly variable.

Mouse models have been developed in order to better understand the relationship between phenotype and genotype in DS. The long arm of this chromosome (21q) was completely sequenced since 2001 [11], and recent transcription comparisons’ studies report that it contains 696 genes, including at least 235 protein-coding genes and 142 pseudogenes, with a large subset of genes which have a mouse homolog located on regions of synteny carried by mouse chromosomes 16, 17 or 10 [12].

Several models carrying additional copies of regions homologous to Hsa21 were generated and used to decipher the contribution of segments to DS phenotypes [3,10]. Locomotor and learning deficits were found associated with trisomy of several segments located on Mmu16: Ts65Dn [13], Ts1Cje [14]; on Mmu17 Ts(17)1Yey [15] or Ts1Yah [16] and on Mmu10 Ts(10)1Yey [15] and in a single gene model for *Dyrk1a* [17–20]. A different model was generated in 2005: the Tc1 transchromosomic mouse line carrying an almost complete copy of Hsa21 with human genes expressed in various tissues [21]. Gribble and al. [22] deciphered the sequence of the Hsa21 present in Tc1 cells, and they identified one deletion, six duplications and 25 de novo structural rearrangements presumably due to the gamma irradiation used during the process of creating the mouse line. Nevertheless, the Tc1 mouse line is the unique humanized model for DS, and displays phenotypes affecting short term memory impairment, the hippocampal function [21] and locomotor activities [23,24].

Further analysis started by combining different models to sort out the contribution of subregions to specific DS phenotypes. The Ts65Dn mouse was crossed to the Ms1Rhr to demonstrate that the Down syndrome critical region previously identified in humans was necessary but not sufficient to induce DS cognitive phenotypes [25–27]. The experiment was carried out again for the *App-Runx1* deletion crossed in Ts65Dn mice, which rescued post-natal lethality and certain cardiac phenotypes [28]. Similarly, monosomy for the region *Cstb-Prmt2* on chromosome Mmu10, named Ms4Yah, was combined with the Tc1 transchromosome to show that the 50 genes orthologous to the Hsa21 region are not involved in Tc1-induced phenotypes [29].

We then further explored the contribution of the *Abcg1-U2af1* region, located on mouse chromosome 17, which contains 14 conserved genes, namely *Abcg1*, *Tff3*, *Tff2*, *Tff1*, *Tmprss3*, *Ubash3a*, *Rsph1*, *Slc37a1*, *Pde9a*, *Wdr4*, *Ndufv3*, *Pknox1*, *Cbs*, *U2af1*, and two additional transcribed units *(loc 102631757* and *AK019514)*. The trisomy of *Abcg1-U2af1* displayed impairment in working memory, in novel object recognition and overexpression of the conserved genes, except *Abcg1* which was inactivated during genetic engineering and *U2af1*, which is located outside the interval [16]. All the genes from *Abcg1-U2af1* genetic interval are trisomic in the Tc1 mouse model except the *Ndufv3* gene which is rearranged [22]. The corresponding monosomy Ms2Yah [30] carries a deletion of the 12 conserved genes, plus the last exons of *Abcg1*, and showed fear conditioning and social recognition defects [30]. To determine whether the region could play a role in several DS phenotypes observed in the Tc1 mouse model, Ms2Yah and Tc1 mouse models were examined for impairments in Open-Field, Morris water maze and rotarod.

## Materials and Methods

### Ethics statement

All animals were treated in compliance with the animal welfare policies of the French Ministry of Agriculture. Yann Herault was granted permission by the French Ministry of Agriculture (law 87 848) under accreditation 67–369. Behavioral experiments were planned in order to evaluate cognition and motor conditions in these mice as described previously in [29], submitted to the local animal care, use and ethic committee of the IGBMC (Com’Eth), and approved under accreditation number (2012–069) to comply with the new regulation in France. Mice were kept under specific pathogen free conditions with free access to food and water for all the tests. The light cycle was controlled as 12 h light and 12 h dark (lights on at 7AM). The Morris water maze (MWM) was conducted between 9:00AM and 1:00 PM. All the other tests were done between 9:00AM and 4:00 PM.

After weaning, male mice were gathered from ten litters and kept as littermates in the same cage with no isolated individual. The different apparatuses used were placed in a dimly lit testing room (approximatively 20 lux). To produce experimental groups, only animals from litters containing a minimum of two male pups were selected. Groups of animals were established for all genotypes on the N2B6C3B genetic background (see below): wt (n = 12), Ms2Yah (n = 11), Tc1 (n = 11), Tc1/Ms2Yah (n = 8). Animals were transferred to the experimental room 30 min before each experimental test. The tests were administered in the following order: open field (week 37), learning and reversal in MWM (weeks 53–56), working memory in MWM (week 62–63), and rotarod (week 72). No invasive procedure was used and the method of euthanasia was CO_2_ inhalation.

### Mouse lines, breeding and genotyping

The Ms2Yah, official name Del(17Abcg1-Cbs)2Yah, mice were generated on 129/Ola ES cells as described previously [16] and backcrossed on the C57BL/6J genetic background at least to N10 in this study [30]. The Tc1 transchromosomic line has been described previously [21]; These mice were kept on an F1 B6C3B background; the C3B are sighted C3H/HeH, a congenic line for the BALB/c allele at the Pde6b gene in C3H/HeH [31]. The two lines were crossed to generate double mutant and control cohorts on a mixed genetic background B6xB6C3B (N2B6C3B) under Specific Pathogen Free conditions.

For the identification of the Ms2Yah allele and the Tc1 chromosome, genomic DNA was isolated from tail biopsies using the NaCl precipitation technique. The Ms2Yah allele was identified using non quantitative PCR with 2 pairs of primers: one control mapping the end of the wild type (wt) allele form of the *U2af1* locus (wt Forward 5-CCAGCTGAAGATGGGTGTGTCTGC-3 / wt Reverse 5-AGCCTTCCCTGGGGACCTGAAA-3) leading to the amplification of a PCR product of 468 bp, and one transgenic pair mapping the junction between the *U2af1* insert [16] and the HPRT 3’ vector (Tg Forward 5-CCAGCTGAAGATGGGTGTGTCTGC-3 / Tg Reverse 5-AACGACCGAGCGCAGCGA-3) amplifying a product of 272 bp. The Hsa21 present in Tc1 mice was identified by PCR using primers D21S55F (5_-GGTTTGAGGGAACACAAAGCTTAACTCCCA-3_) and D21S55R (5_-ACAGAGCTACAGCCTCTGACACTATGAACT-3_) that are specific for the Hsa21 and control primers specific for the mouse genome (MyoF: 5′-TTACGTCCATCGTGGACAGCAT-3′, MyoR: 5′-TGGGCTGGGTGTTAGTCTTAT-3′) with specific PCR products of 208 bp and 245 bp, respectively.

### Open field

This test measures rodent behavioral responses such as locomotor activity, hyperactivity and exploratory behaviors within a closed space. The test was carried out in a 55 cm diameter round white box and mouse activity was recorded with a video tracking system (Ethovision, Noldus, France) during a single 30 min session. After each mouse trial, the arena was thoroughly cleaned with 50% ethanol, followed by one cleaning with water, and then dried with paper towels to minimize olfactory cues. We quantified the speed and distance traveled during 3 phases (0 to 10 min; 10 to 20 min and 20 to 30 min). We also measured the percentage of time spent in each arena zone (peripheral, intermediate, central) in the same phases of the session.

### Morris water maze spatial memory

**Learning protocol.** The water maze was a circular pool (150-cm diameter, 60-cm height) filled to a depth of 40 cm with water maintained at 20°C–22°C, made opaque using a white aqueous emulsion (Acusol OP 301 opacifier) and split into 4 quadrants: South-East (SE), North-West (NW), North-East (NE), South-West (SW). The escape platform, made of rough plastic, was submerged 1 cm below the water’s surface.

This experiment was performed to study reference memory through a spatial search strategy that involved finding a hidden platform (6 cm diameter) in a pool. In the reversal mode mice had to learn a new platform position. All the procedures were adapted from Morice et al., 2008 [24] and Duchon et al., 2011 [29]. The spatial memory session consisted of a 6-day (S1 to S6) learning phase with two 90-second trials per day. Each trial started with mice facing the interior wall of the pool and ended when they climbed onto the platform located on the SE quadrant or after a maximum searching time of 90 sec. The starting position was changed pseudo-randomly between trials. Mice were left undisturbed in their home cage for 90-min inter-trial intervals. On the seventh day, mice were given the 60-sec probe test in which the platform had been removed. The distance traveled in each quadrant (NW, NE, SW, SE) was recorded to quantify the time spent in the target quadrant.

**Reversal Learning protocol.** After the first probe trial, all mice were given a reversal test, in which the hidden platform was moved to a new position (NW,). Mice were trained for 5 days (Reversal Session RS1 to RS5) following the same training procedure and then tested for the second probe trial on the sixth day (13 days after the beginning of the total test). To test mice for long term memory, they were left undisturbed for 20 days before being given a third probe trial.

### Rotarod

The Rotarod test was performed to estimate rodent locomotor coordination. The apparatus (Bioseb, France) is made of a rotating bar 5 cm in diameter (hard plastic materiel covered by grey rubber foam) on which mice are placed facing the direction of rotation. The first phase was a learning period composed of one training session with 4 trials per day for 3 days. For each trial mice were placed on a rotating rod, starting from 0 and accelerating to 40 rpm in 5 minutes. We recorded the time spent on the rod and the speed before the fall. The second step of the task occurred on the 4^th^ day. It consisted of 7 trials of 2 minutes, each trial being performed at one selected speed (4, 10, 16, 22, 28, 34 and 40 rpm). This procedure was repeated twice and the time spent on the rod was recorded for each trial.

### Statistical analyses

ANOVA was performed to analyze differences between the 4 genotype groups using dedicated commercial Software (Sigmaplot): we applied a two way ANOVA for the Open-Field, Morris Water Maze Probe trials’ and rotarod test phase. we used a two Way repeated measures ANOVA for Learning/Reversal protocols and the learning Rotarod phase. However, for all the ANOVA, the post hoc analysis was done using Tukey’s method. Data are presented as mean ± s.e.m.

## Results

### The exploratory pattern in the open field is not altered in Tc1/Ms2Yah mice

**Figure 1:**
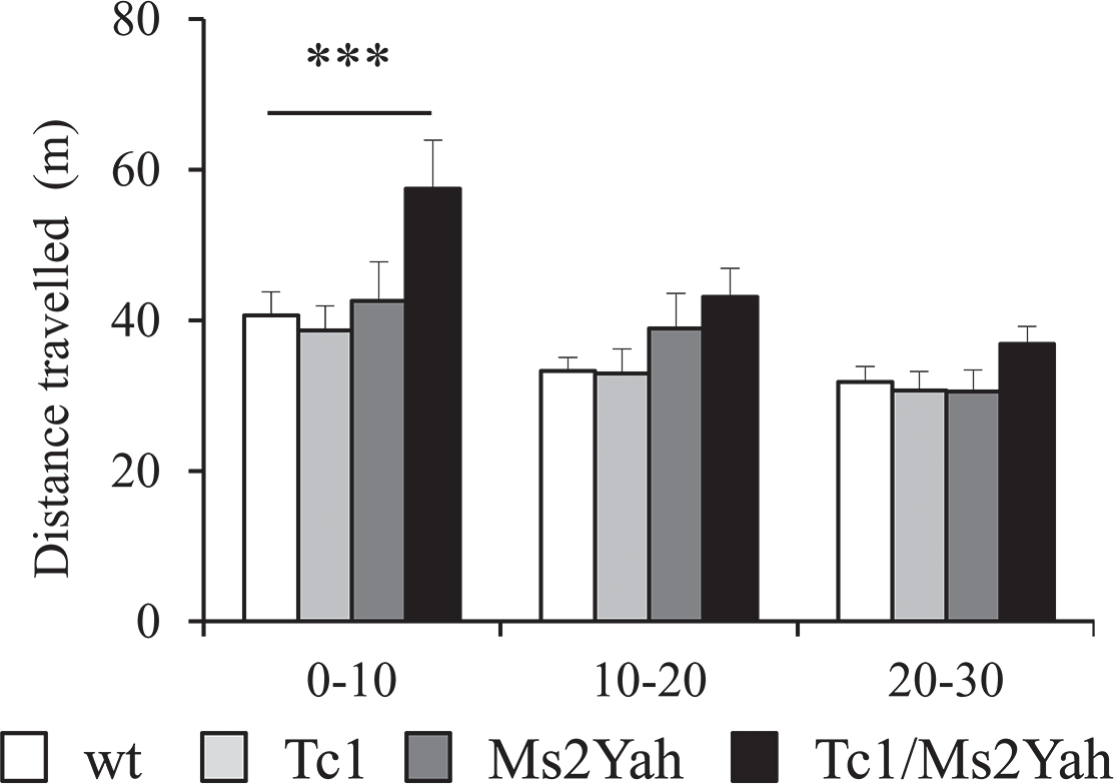
Open field locomotor activity of mice. The distance travelled (m) during the exploration of the open-field by mice with different genotypes are shown Tc1/Ms2Yah during the consecutive 0–10, 10–20, 20–30 min intervals. The activity of Tc1/Ms2Yah was increased mainly during the first 10 minutes (parameter “distance travelled”, Two-way ANOVA “0–10 min”, F(2,111) = 12.524 p < 0.001; Tuckey’s post hoc method “wt vs Tc1/Ms2Yah” q = 4.663, p = 0.007; “Ms2Yah vs Tc1/Ms2Yah” q = 5.108, p = 0.003 and “Tc1 vs Tc1/Ms2Yah” q = 3.968, p = 0.03Tc1/Ms2YahTc1/Ms2Yah). Values are means + s.e.m.

As described previously the Tc1 mouse displayed a hyperactive exploratory phenotypes in the B6129 [23] but not in the B6C3B background [29]. In order to evaluate exploratory behavior, locomotion deficits and increased anxiety related behavior, we used the open field and we measured horizontal activity during three consecutive 10-minutes intervals (Fig. 1). Habituation was observed for all the genotypes (Two-way ANOVA “time intervals” F(2,111) = 12.524 p < 0.001; Tukey’s post hoc test “0–10 min vs 20–30 min” q = 7.001, p < 0.001) and exploration in the center versus the periphery was similar with no anxiety pattern in the Tc1 group or in Tc1/Ms2Yah (data not shown). The total distances travelled are globally the same after thirty minutes. However, during the first ten minutes, the Tc1/Ms2Yah group are more active compared to control and monosomic individuals (Fig. 1; parameter “distance travelled”, Two way ANOVA “time intervals 0–10 min”, F(2,111) = 12.524 p < 0.001; Tuckey’s post hoc method “wt vs Tc1/Ms2Yah” q = 4.663, p = 0.007; “Ms2Yah vs Tc1/Ms2Yah” q = 5.108, p = 0.003 and “Tc1 vs Tc1/Ms2Yah” q = 3.968, p = 0.03). This slight change in the initial exploratory phase in the Tc1/Ms2Yah double-mutants suggested that the *Abcg1-U2af1* region might be involved in controlling exploratory behavior. Nevertheless, this behavior was not found in the Tc1 genotype mice, unraveling a hyperactive phenotype in the Tc1 mutant mice on this N2B6C3B background.

### Memory impairment in the Morris watermaze of Tc1 mice depends on the number of copies of the *Abcg1-U2af1* region

**Figure 2:**
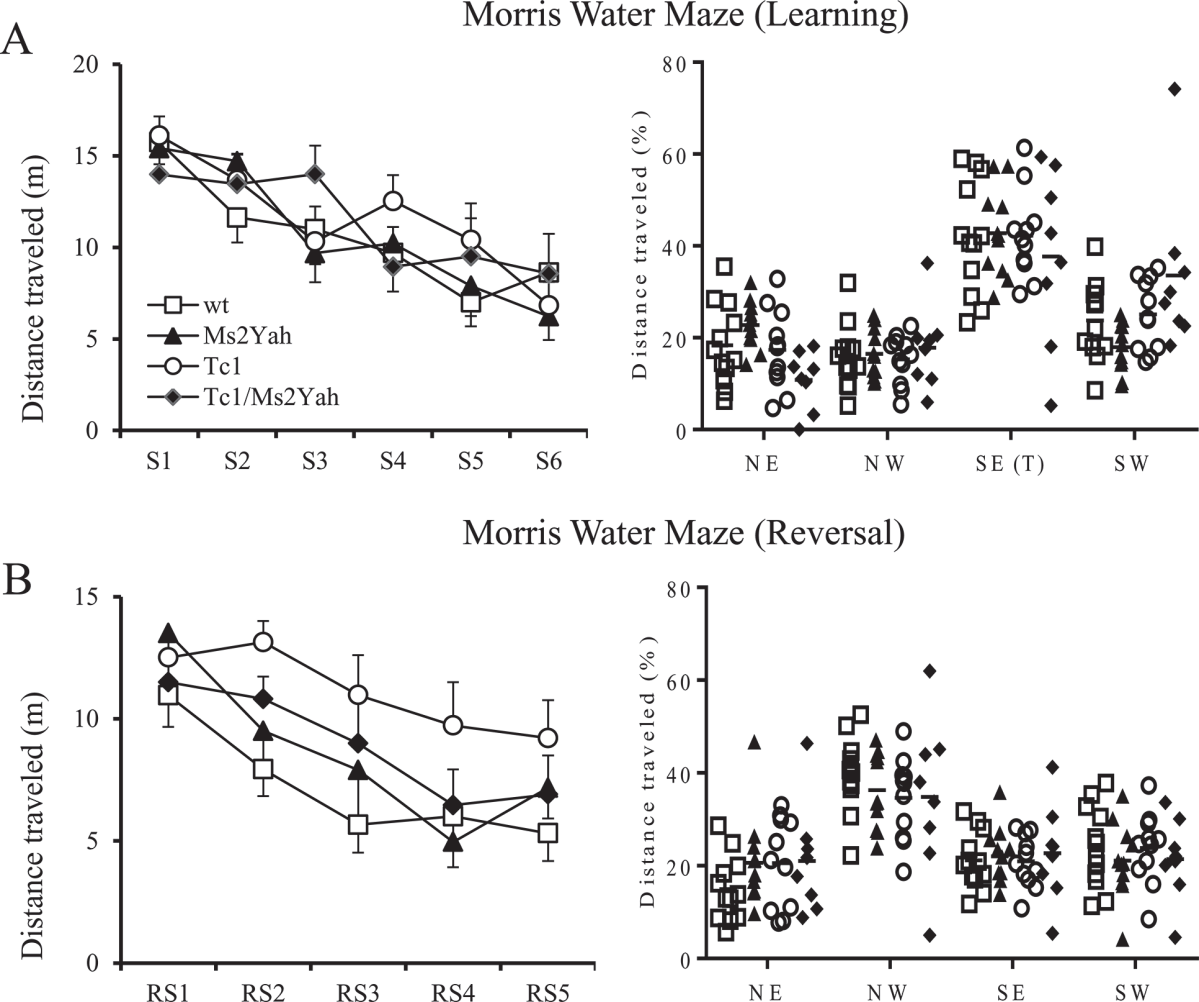
Learning and Reversal reference memory performance of mice in the Morris water maze. (A) The distance traveled on left panel (m) and the percentage of distance traveled in the target quadrant (SE) during the probe trial (S7) on the right (with data from every individual shown), are shown during learning in the Morris water maze. Mice from the four genotypes learned where the platform is located (SE) with a reduction of the distance traveled to find the platform over the 6 learning sessions (S1 to S6). In the right panel, the horizontal line indicates the distance travelled using a random search strategy 25%. No difference was observed for the different genotypes. (B) In the consecutive reversal session we observed similar learning capacities when the platform position was located in the NW quadrant, and after five reversal learning days (RS1 to RS5) shown on the left. During the following probe trial (right panel, RS6), individuals from all the genotypes spent more time in the new target quadrant (NW). Values represent mean + s.e.m.

To evaluate the impact of the *Abcg1-U2af1* region on learning potential and memory, we performed a Morris water maze test. During the first phase for place learning (Fig. 2A), all four genotypes found the platform with similar efficiency and memorized where the platform was located (parameter “distance travelled”, two way repeated measures ANOVA “learning day”, F(1,38) = 64.517 p < 0.001). The distance traveled was reduced between the first and the last day of learning for all genotypes (Two-way repeated measures ANOVA, Tukey’s post hoc test “S1 vs S6”, wild type q = 5.643, p < 0.001; Ms2Yah q = 6.973, p < 0.001; Tc1 q = 7.037, p < 0.001, Tc1/Ms2Yah q = 3.513, p < 0.001). In the probe test (Fig. 2A right panel, 24 hours after the learning session, all mice traveled further in the target quadrant than in the rest of the arena (parameter “% distance travelled” two way ANOVA “quadrant”, F(1,160) = 133.77 p < 0.001)) and no genotype effect was detected (Tukey’s post hoc method “target quadrant vs non target quadrants”, wild type q = 9.322, p < 0.001; Ms2Yah q = 9.301, p < 0.001; Tc1 q = 9.002, p < 0.001, Tc1/Ms2Yah q = 5.678, p < 0.001). The spatial learning and memory of the Tc1 and Tc1/Ms2Yah mice was not affected compared to wild type mice.

During the second phase, the reversal phase, all genotypes showed significant global learning between RS1 and RS5 (Fig. 2B; Two way repeated measures ANOVA “trials”, “genotype”, F(1,38) = 33.018, p < 0.001), but the performance of the Tc1 group are reduced compared to wild type and Ms2Yah (Tukey’s post hoc method, Tc1 RS1 vs RS5, ns). This phenotype was rescued in Tc1/Ms2Yah compound mice (Tukey’s post hoc method: q = 3.322, p = 0.024). One day after the last reversal session, a probe trial was performed (Fig. 2B right panel) and we found that all mice remembered the correct position of the platform with greater distance travelled in the target quadrant (parameter “% distance travelled” two way ANOVA “quadrant”, F(1,160) = 88.878 p < 0.001, Tukey’s post hoc method “target quadrant vs non target quadrants”, wild type q = 9.383, p < 0.001; Ms2Yah q = 6.830, p < 0.001; Tc1 q = 5.840, p < 0.001, Tc1/Ms2Yah q = 5.088, p < 0.001). None of the groups displayed memory deficits since they described the same performance during the 2 probe trials. However, Tc1 mice needed more time to learn the new task.

### Deficits in motor coordination of the Tc1 mice are partially rescued by the loss of *Abcg1-U2af1* trisomy

**Figure 3:**
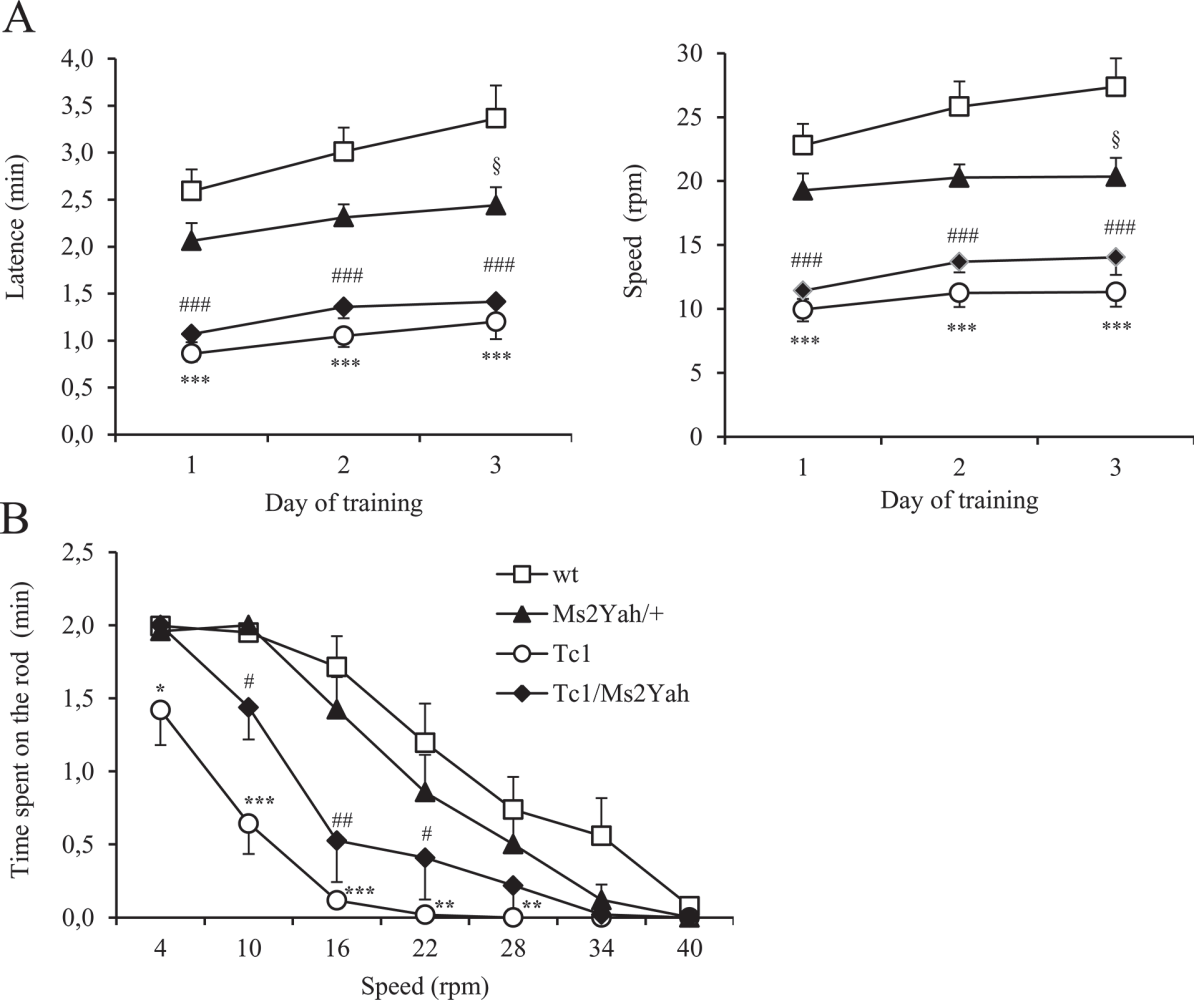
Locomotor performance through accelerating and continuous speed rotarod tasks. (A) We examined locomotor coordination through three days of learning an acceleration protocol (from 4 to 40 rpm over 5 min). Results are expressed as the time (minutes) that mice remained on the rod before falling (left panel), and the velocity at the time of falling (right panel). Although Ms2Yah did not express any deficit, mice could not improve their performance over the 3 days (figure A, left panel: § p = 0.376). Conversely, Tc1 mice were unable to stay on the rod longer, or improve their time over the 3 days (figure A, left panel: *** p<0.001 between wt and Tc1 and ### p<0.001 between wt and Tc1/Ms2Yah groups). (C) The graph displays the time (min) that mice stayed on the rotarod during the test phase when the speed was set at 4, 10, 16, 22, 28, 34 and then 40 rpm, for a maximum of 2 minutes. The performance of the Tc1 group was poor from the beginning of the task (Two-way ANOVA “wt vs Tc1” on speed 4rpm* p<0.05, 10/16 rpm *** p<0.001) and 22/28 rpm ** (p<0.01). Interestingly, the performance of Tc1/Ms2Yah mice was in-between that of Tc1 and controlsand the Ms2Yah did not show major differences compared to wt. Values represent means + S.E.M.

Mutant mice were screened for motor skill using the rotarod test, as described previously [29]. Two patterns were observed during the training phase: wild type and Ms2Yah mice displayed the same capacities to stay on the rod, whereas both Tc1 and Tc1/Ms2Yah mice spent less time on the rod than the wild type control (Fig. 3A; repeated ANOVA “genotype” Tukey’s post hoc analysis; F(3.64) = 25.932. p < 0.001; wild type vs Tc1: q = 11.180. p < 0.001; wild type vs Tc1/Ms2Yah: q = 9.133. p < 0.001) and Ms2Yah littermates (Ms2Yah vs Tc1. q = 7.066. p < 0.001. Ms2Yah vs Tc1/Ms2Yah. q = 5.297. p = 0.004) over the 3 days during the learning phase. Moreover, while the wild type improved their performance during the training period (ANOVA “days”, Tukey’s post hoc method: F(2.64) = 11.477, p < 0.001; wild type day3 vs day1, q = 6.014, p < 0.001), none of the other groups, Ms2Yah, Tc1 and Tc1/Ms2Yah, progressed at the end of the training.

During the test phase (Fig. 3B), we determined the time spent on the rod at a given constant speed, in two sessions per trial and with increasing speed between each trial (4, 10, 16, 22, 28, 34 and 40 rpm) for a maximum of 2 min. As with the training session, a significant difference was observed in performance between the wild type and Ms2Yah versus the Tc1 and Tc1/Ms2Yah genotypes while the speed increased from 4 rpm to 34 rpm (Two way ANOVA “speed” and “genotype”; Tukey’s post hoc method: F(18,224) = 3.308, p < 0.001; wt vs Ms2Yah q = 3.493, p = 0.06; wt vs Tc1 q = 15.000, p < 0.001; wt vs Tc1/Ms2Yah q = 8.389, p < 0.001; Ms2Yah vs Tc1 q = 11.599, p < 0.001; Ms2Yah vs Tc1/Ms2Yah q = 5.219, p < 0.001). An intermediate behavior was detected in Tc1/Ms2Yah mice with better exercise performance than Tc1 but lower than wild type mice (at 10 rpm, wt vs Tc1/Ms2Yah q = 8.923, p<0.001; Ms2Yah vs Tc1/Ms2Yah ns; Tc1 vs Tc1/Ms2Yah q = 4.766, p = 0.004 - at 16 rpm, wt vs Tc1/Ms2Yah q = 10.514, p<0.001; Ms2Yah vs Tc1/Ms2Yah q = 5.502, p<0.001). In this experiment, Ms2Yah mice showed normal coordination, but could not increase their performance on the rod during the training days; the *Abcg1-U2af1* region might play a role in locomotor abilities because, although training appeared equivalent to that of wild type mice, a longer test session revealed that double mutants Tc1/Ms2 could stay longer on the rod than Tc1 mice.

## Discussion

The present study highlights the contribution of the *Abcg1-U2af1* genetic interval to DS-related features in Tc1 mouse models. We found that reducing the genetic dosage of this region in the Tc1 mouse models rescued subtle impairments in reversal learning, working memory and did so partially in the rotarod test, but had no impact on hyperactivity or spatial learning.

### Robustness of Tc1 induced deficits and the influence of the genetic background

In this third study focusing on the behavior phenotype of the Tc1 DS mouse model, we performed the analysis in a new mixed genetic background, i.e. N2B6C3B. Originally, the Tc1 model was studied in the B6129S8 [23,24,32] and more recently transferred in the B6C3B genetic background [28,29,33]. We generated the Ms2Yah, carrying the deletion of the *Abcg1-U2af1* region, on the C57BL/6J background. Thus when we crossed B6.Ms2Yah with the Tc1 transchromosomic line kept in the B6C3B genetic background, the Tc1/Ms2Yah compound mice and the relative control groups, wild-type, Ms2Yah and Tc1, were on the N2B6C3B background. Tc1 mice are known to be mosaic, with some cells losing the Hsa21 chromosome [21]; nevertheless, we recapitulated most of the Tc1 phenotypes (Table 1) in the MWM tests (for spatial learning), and in the rotarod tests (learning phase and challenge tests). The hyperactive phenotypes observed in the 129S8 [23], was not found in the B6C3B background [29] but was replicated again in the N2B6C3B background (this study). We hypothesize a contribution of the B6 genetic background which displays a higher level of locomotor activity than 129 and C3H [34–36]. Thus, even if most of the Tc1-induced phenotypes are robust, a few are also affected by the genetic background. The set of disomic genes of the different genetic backgrounds modified the Tc1-induced phenotypes. This study proposes that the variability in the expressivity and penetrance of the features found in DS people depends on genetic interactions between the trisomic genes and the whole genome.

**Table 1:** Robustness of the Tc1 induced phenotypes observed in different genetic backgrounds and interference with different rescues.

Mouse Models	Tc1^*^	Tc1^§^	Tc1/ Ms4Yah^§^	Tc1^§^	Tc1/ Ms2Yah^§^	Ms2Yah^§^
Background	B6129	B6C3B	B6C3B	N2F1	N2F1	N2F1
Hyperactivity (open field)	+	=	=	+	+	=
MWM spatial learning and memory	=	=	=	=	=	=
MWM reversal learning and memory	=	=	=	-	=	=
Rotarod learning	-	-	-	-	-	=
Rotarod test	-	-	-	-	±	=
						

(+), (=), (−) or (±) respectively indicate a reported effect with an improvement, similar or with an impairment compared to the control littermates. The (±) corresponds to a partial rescue compared to the transchromosomic Tc1 model. Data adapted from [23,24] ^*^, [29] ^§^ and this work.

### The *Abcg1-U2af1* region contributes to learning and memory deficits in the Tc1 DS mouse models

Here we found that reducing the number of copies of the mouse genes located in the *Abcg1-U2af1* region, restores some deficits of the Tc1 model in the reversal phase of MWM. The Tc1/Ms2Yah mice learned the location of the platform with similar efficiency to controls while the Tc1 mice needed more sessions to do so. Although all the genotypes finally learned where the platform was located in the probe test, the decrease in the performance of the Tc1 group could be associated with a lack of cognitive flexibility, since learning memory was not altered. This particular phenotype is observed in Down syndrome people [37] and our results suggest that an increase in one or more genes of the *Abcg1-U2af1* region contributes to decreased behavioral flexibility.

Tc1 mice have severe deficits in motor skills in different motor coordination tasks such as rotarod, static rod and footprint tests [23]. Rotarod performance analysis in our study confirmed the deficit in the locomotor activity of Tc1 mice, which occurred in the training days and in the test phase. After consecutive days of training, mice usually enhance their performance by staying on the rod longer each day [38,39]. In our experiment, Tc1 and Tc1/Ms2Yah did not improve their performance in the learning phase. The learning mechanisms of locomotor function were altered, and decreasing the number of copies of the *Abcg1-U2af1* region could not rescue them.

During the test phase with different increasing rotarod speeds, Tc1 mice displayed strong impairment compared to controls. The Tc1/Ms2Yah showed better performance by staying longer and at higher speed on the rod than the Tc1 group. We exclude a strong contribution of the genetic background to this phenotype since the C3H fell earlier than the B6 in a similar protocol and the learning phase was not compensated [6]. Thus, even if the Ms2Yah region is not implicated directly in motor learning, at least the over-expression of one or more genes located in the interval definitely modifies locomotor activity.

### Searching for candidates in the *Abcg1-U2af1* genetic interval

Identifying genes in the *Abcg1-U2af1* region modulating Tc1-induced locomotor phenotypes would certainly improve our understanding of DS and stimulate further therapeutic approaches. As a consequence of the results described here, candidates should be conserved between humans and mice and expressed during the development of the adult brain. Many regions of the brain are involved in motor learning and performance, such as the cerebellum, basal ganglia, and the motor cortex. Thus expression of genes in the brain might help to discriminate candidates for the different phenotypes described here. According to the Allen brain atlas, *Abcg1*, *Tff1*, *Ubash3a*, *Pde9a*, *Ndufv3*, *Pknox1* and *Cbs* were found expressed in the adult cerebellum and in the isocortex and are thus candidates. Of particular interest are *Pde9a* and *Cbs*, which are both expressed in the central nervous system. Pde9a codes the phosphodiesterase 9a and transforms cAMP and cGMP into their respective monophosphate forms and its inhibition can stimulate neuronal plasticity [40,41]. A delay in neuronal transmission may partially explain the change in learning tasks. Otherwise, the *Cbs* gene encodes cystathionine-beta-synthase whose deficiency causes homocystinuria (OMIN236200), a metabolic disorder with intellectual disabilities. Cbs is a strong candidate for phenotypes described by both monosomic and trisomic models in the hippocampus [16,30]. In addition, a mouse model overexpressing human CBS displayed an increase magnitude of the long term potentiation in vitro similar to the Ts1Yah electrophysiological phenotypes observed in vivo [42].

Only a few cases of DS have been reported with partial trisomy overlapping the most telomeric part of human chromosome 21 [43,44] and so far none have been described with a trisomy limited to this segment. Conversely several cases with monosomy of the telomeric end have been described with mild phenotypes [45–48]. Similarly, analysis of trisomy and monosomy models for *Cstb-Prmt2* showed no phenotypes in open field, in the Morris water Maze, either alone or in combination with the Tc1 transchromosome [15,29,49]. Only in fear conditioning task, the Df(10)Yey/- displayed a deficit in contextual memory [49].

In this report, we confirmed that rescuing the number of copies of *Abcg1-U2af1* modulates Tc1-induced phenotypes only slightly, although the region was sufficient alone to induce certain learning and memory deficits [15,16], and as such this genetic interval certainly contributes, along with other regions of Hsa21, to the variability of DS features.
